# Genetic Variant in *GRM1* Underlies Congenital Cerebellar Ataxia with No Obvious Intellectual Disability

**DOI:** 10.3390/ijms24021551

**Published:** 2023-01-12

**Authors:** Maria S. Protasova, Tatiana V. Andreeva, Sergey A. Klyushnikov, Sergey N. Illarioshkin, Evgeny I. Rogaev

**Affiliations:** 1 Vavilov Institute of General Genetics, Russian Academy of Sciences, 119991 Moscow, Russia; 2 Center for Genetics and Life Science, Department of Genetics, Sirius University of Science and Technology, 354340 Sochi, Russia; 3 Centre for Genetics and Genetic Technologies, Department of Genetics, Faculty of Biology, Lomonosov Moscow State University, 119192 Moscow, Russia; 4 Department of Neurogenetics, Research Center of Neurology, 123367 Moscow, Russia; 5 Department of Psychiatry, UMass Chan Medical School, Shrewsbury, MA 01545, USA

**Keywords:** cerebellar ataxia, cerebellar hypoplasia, early-onset ataxia, metabotropic glutamate receptor 1, *GRM1*, mGluR1

## Abstract

Metabotropic glutamate receptor 1 (mGluR1) plays a crucial role in slow excitatory postsynaptic conductance, synapse formation, synaptic plasticity, and motor control. The *GRM1* gene is expressed mainly in the brain, with the highest expression in the cerebellum. Mutations in the *GRM1* gene have previously been known to cause autosomal recessive and autosomal dominant spinocerebellar ataxias. In this study, whole-exome sequencing of a patient from a family of Azerbaijani origin with a diagnosis of congenital cerebellar ataxia was performed, and a new homozygous missense mutation in the *GRM1* gene was identified. The mutation leads to the homozygous amino acid substitution of p.Thr824Arg in an evolutionarily highly conserved region encoding the transmembrane domain 7, which is critical for ligand binding and modulating of receptor activity. This is the first report in which a mutation has been identified in the last transmembrane domain of the mGluR1, causing a congenital autosomal recessive form of cerebellar ataxia with no obvious intellectual disability. Additionally, we summarized all known presumable pathogenic genetic variants in the *GRM1* gene to date. We demonstrated that multiple rare variants in the *GRM1* underlie a broad diversity of clinical neurological and behavioral phenotypes depending on the nature and protein topology of the mutation.

## 1. Introduction

Glutamate is the essential neurotransmitter in certain parts of the central nervous system, one of which is the cerebellum. There are several types of glutamate receptors: ionotropic glutamate receptors (iGluRs) mediated fast synaptic transmission and metabotropic glutamate receptors (mGluRs), which are coupled to G proteins and produce a more complex postsynaptic response consisting of both internal calcium release and a slow excitatory postsynaptic potential [1]. mGluRs include eight types of receptors, constituting three groups depending on their location and functions [2]. Group I includes mGluR1 and mGluR5, which are located predominantly postsynaptically and involved in the modulation and plasticity of excitatory synaptic transmission via Gq/G11 proteins and ligand binding activation of phospholipase Cβ leading to phosphoinositide hydrolysis [3]. Group II (mGluR2 and mGluR3) and group III (mGluR4, mGluR6, mGluR7, and mGluR8) are found on presynaptic and postsynaptic membrane and coupled to Gi/o proteins; their activation leads to inhibition of adenylyl cyclase [2]. mGluR1 is encoded by the *GRM1* gene, which has the highest expression level in the cerebellum from early development and during ontogenesis [4,5]. However, the expression is not limited to Purkinje cells, and the *GRM1* gene is also expressed in the cerebral cortex, basal ganglion, medulla oblongata, spinal cord, heart, bone marrow, and kidney. Alternative splicing of the *GRM1* gene transcript results in the formation of four mGluR1 isoforms. These isoforms have identical extracellular regions, consisting of a venus flytrap domain and cysteine-rich domain, seven transmembrane domains, and differ by distinct C-termini [6,7]. In Purkinje cells, mGluR1α and β are expressed. mGluR1α is the major longest isoform that is formed from dimers and localized on dendritic spines of Purkinje cells. mGluR1β have intracellular localization; however, they can be transported to the postsynaptic membrane and form heterodimers with mGluR1α [8]. Purkinje cells receive two distinct excitatory inputs from parallel and climbing fibers [9]. As the result of multiple stimuli from presynaptic terminals, the superfluous release of glutamate leads to the activation of mGluR1-mediated slow synaptic transmission and long-term depression [10]. Impaired mGluR1 signaling is found in different spinocerebellar ataxias in humans and many animal models of cerebellar ataxia [11]. 

Cerebellar hypoplasia is a rare brain pathology that results in a severe disorder, most often from birth, characterized by psychomotor developmental delay, ataxia, and in most cases, accompanied by intellectual disability [12,13]. Cases of cerebellar hypoplasia without cognitive impairment and the involvement of other organs and tissues are extremely rare. Previously, we established the heterogeneity of X-linked and autosomal recessive diseases which are characterized early-onset cerebellar ataxia without cognitive impairment. In several familial cases of different ethnic origins with clinically similar phenotypes, mutations were identified at different evolutionarily highly conserved genetic loci. In the first case, a missense mutation in the *ABCB7* gene was identified in a large Buryat family, which led to X-linked recessive cerebellar hypoplasia [14,15]. In the second case, two brothers with a diagnosis of congenital cerebellar ataxia and generalized polyneuropathy were found to have a mutation in the *LRCH2* gene, the role of which is not clearly known, but it is highly expressed in the embryonic brain, especially in the cerebellum and brain regions involved in motor functions [16]. Furthermore, another genetic locus was detected in four patients with a diagnosis of early-onset cerebellar ataxia from two unrelated families in the North Caucasus region. This mutation in the *CSMD1* gene, which is involved in brain development and the growth of neuronal processes, has a founder effect [16]. 

In this study, we performed a genetic analysis in the case of autosomal recessive cerebellar ataxia with no obvious intellectual disability. A novel mutation has been identified in the metabotropic glutamate receptor 1 gene (*GRM1*) that plays an important role in the development of the brain during the embryonic period. Mutations in the *GRM1* gene are extremely rare. The discovered mutation expanded the range of genetic defects in the *GRM1* gene underlying a variety of clinical manifestations.

## 2. Results and Discussion

The disease in a patient from an Azerbaijani family (AIV-1) was diagnosed from birth. The patient’s parents were healthy. From infancy and early childhood, there was a delay in physical development. He began to walk at around the age of 4–4.5 years, but from the beginning, walking was awkward, and he often stumbled. From about 6–7 years of age, the condition remained relatively stable. The patient studied at a special boarding school. Medical examination at the Research Center of Neurology was carried out in the third decade of life. The following neurological symptoms were revealed: dysarthria, truncal ataxia during standing and walking, and coarse discoordination when performing dynamic tests in the hands. MRI showed hypoplastic and underdeveloped cerebellar hemispheres, enlarged fourth ventricle, and cisterna magna due to hypoplasia of the caudal vermis and paramedian zones of the cerebellar hemispheres, with normal corpus callosum, basis pontis, and medulla. The patient was diagnosed with congenital cerebellar hypoplasia. According to the results of a general examination and a survey of relatives, no obvious intellectual disability has been observed from early development to the present. When tested with the Mini-Mental State Examination (MMSE) scale, the patient scored 27 points, which formally fits into the category of “mild cognitive decline.” However, it should be noted that due to certain difficulties with the Russian language (in everyday life, the patient speaks to people mainly in Azerbaijani), cognitive testing was carried out by Russian-speaking medical personal with the participation and help of a Russian-speaking adult relative. Such examination conditions made it difficult to communicate and correctly evaluate the test results. On repeated testing, the performance was better, which was in line with the norm. These results were strikingly different from those observed in other patients with cerebellar hypoplasia and intellectual disability described in the literature. In those cases, there was usually a pronounced cognitive defect with a decrease in the patient’s adaptation to everyday life and the need for outside help [12,13]. Thus, in our patient described in the article, one can rather talk about minor neurodynamic changes and the preservation of the cognitive sphere. From all the above, it can be concluded that the patient had no clinically relevant cognitive impairments.

**Figure 1:**
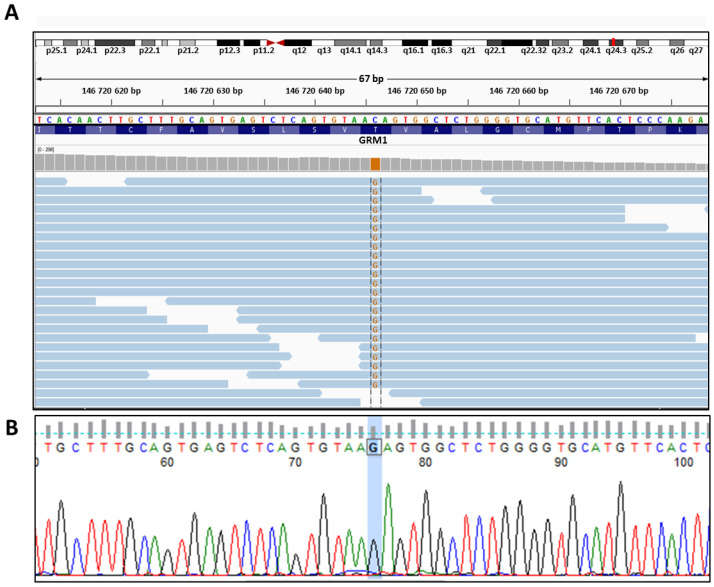
Identification of a novel mutation in the *GRM1* gene in a patient AIV-1. (**A**)—Visualization of the genomic region harboring the candidate non-synonymous variant in the *GRM1* gene (hg19 chr6:g.146720646 C>G) using the IGV tool [29]. (**B**)—Validation of a candidate homozygous variant in patients (AIV-1).

Genetic analysis was performed using whole-exome sequencing of one patient AIV-1. All rare variants with a low global minor allele frequency (gMAF < 0.01) in the 1000 Genomes Project and gnomAD databases were selected [17,18]. Additionally, homo- and hemizygous variants present in healthy individuals in our data collection were filtered. Next, we searched for the strongest mutation that corresponds to the criteria of highly conservative site, “deleterious” prediction, and high level of gene expression in the developing cerebellum. The missense mutation in the *GRM1* gene was identified in patient, AIV-1 (Figure 1A). We conducted large-scale analyses using different databases. This mutation is absent in all 2.5 thousand individuals from 26 populations of 1000 Genomes Project phase 3. This mutation is absent in all 141.456 thousand individuals from gnomAD v2.1 (including 60.146 thousand controls and 114.704 thousand non-neuro) and all 76.156 individuals from gnomAD v3.1.2 (including 14.456 thousand controls, 67.442 thousand non-neuro and 158 individuals with Middle Eastern origin). The Azeri people belong to Turkic ethnic groups. Therefore, we searched for this mutation in various databases of genetic variants from people related to the geographic region of Turkic ethnic groups. We did not find this mutation in 2.497 thousand individuals from the Greater Middle East (GME) Variome Project [19]. There is no mutation in the Iranome database consisting of whole exome data of 800 individuals from eight major Iranian ethnic groups, one of which is Iranian Azeris (24%) [20]. Moreover, this mutation is absent in dbSNP, ClinVar, and HGDM databases [17,18,21,22,23,24]. The variants in the *GRM1* gene are a known cause of spinocerebellar ataxia 44, SCA44, (OMIM 617691) and spinocerebellar ataxia autosomal recessive 13, SCAR13, (OMIM 614831). The found mutation is located in an evolutionarily highly conservative region; the PhyloP score of this site is 7.7842. SIFT and PolyPhen2 programs predicted that this variant is “deleterious” (0.01) and “probably damaging” (0.999) [25,26]. CADD predicted a score of 26.7 which means it was among the 1% most deleterious substitutions in the human genome [27,28]. The mutation in the *GRM1* gene was confirmed by Sanger sequencing (Figure 1B).

**Figure 2:**
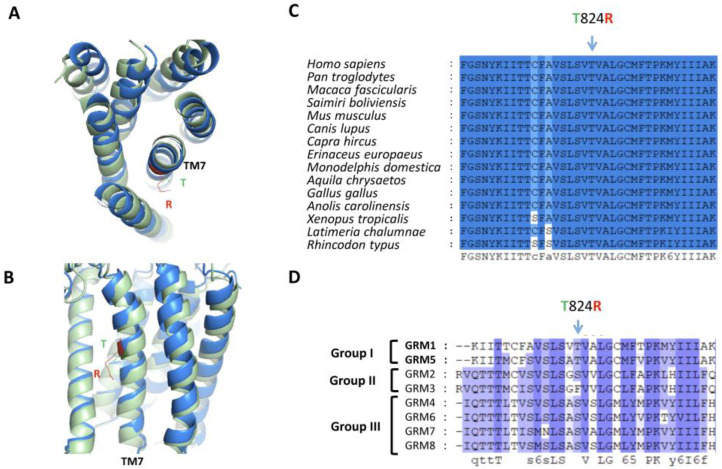
Analysis of p.Thr824Arg (T824R) amino acid substitution effect on the mGluR1 protein structure. (**A**,**B**)—Alignment of the spatial structure of the wild-type (green) and mutated (blue) transmembrane domain containing the p.Thr824Arg amino acid substitution in a patient AIV-1. Protein spatial structure predicted by Phyre2 [33]. (**C**,**D**)—Evolutionary analysis of the conservation of the mGluR1 protein region among orthologs (**C**) and paralogs (**D**).

The nucleotide substitution C>G in the *GRM1* gene leads to a change in the polar uncharged amino acid threonine with an electrically positively charged amino acid arginine at position 824 (p.Thr824Arg) in both long (alpha) and short (beta) isoforms of the glutamate metabotropic receptor 1 (mGluR1) protein. The amino acid threonine is located in a large, evolutionarily highly conserved region and is present in all analyzed orthologues from cyclostomes to primates (Figure 2 and Figure S1). Threonine is also conserved in both mGluR5 isoforms, which, together with mGluR1, belong to group I of postsynaptic glutamate metabotropic receptors that increase NMDA receptor activity [30]. In other metabotropic glutamate receptors, there is serine in this position, which has a similar structure to threonine, with the exception of the metabotropic glutamate receptor mGluR3, which has an uncharged hydrophobic amino acid phenylalanine in this position. This amino acid substitution occurs in transmembrane domain 7 (TM7), which forms a metabotropic channel. MutationTaster2021 predicted this variant as “Deleterious” with a Grantham Matrix Score of 71 and potential loss of TM7 [31]. The complete removal of TM7, as a result of a 3 bp deletion in exon 8 in combination with a mutation in the splicing site of intron 8, leads to disruption of the mGluR1 structure and the autosomal recessive form of SCAR13, which was previously described in a family from Rome [32]. The Phyre2 prediction did not find any alteration in the alpha chain of the secondary structure of wild-type and mutated proteins [33]. However, TM7 of mGluR is necessary for ligand binding, which modulates signaling by acting as positive or negative allosteric modulators [7,34,35,36]. The amino acid arginine, compared to threonine, has a long electrically positively charged chain, which can form excess hydrogen or ionic bonds and, presumably, can interfere in functional interactions with the ligand. No other missense mutations have been reported previously in this domain. The location of the mutation may explain the specificity of the endophenotype (cerebellar hypoplasia without intellectual disability), while all other reported cases for autosomal recessive ataxia linked to *GRM1* mutations were also characterized by severe cognitive impairments.

**Figure 3:**
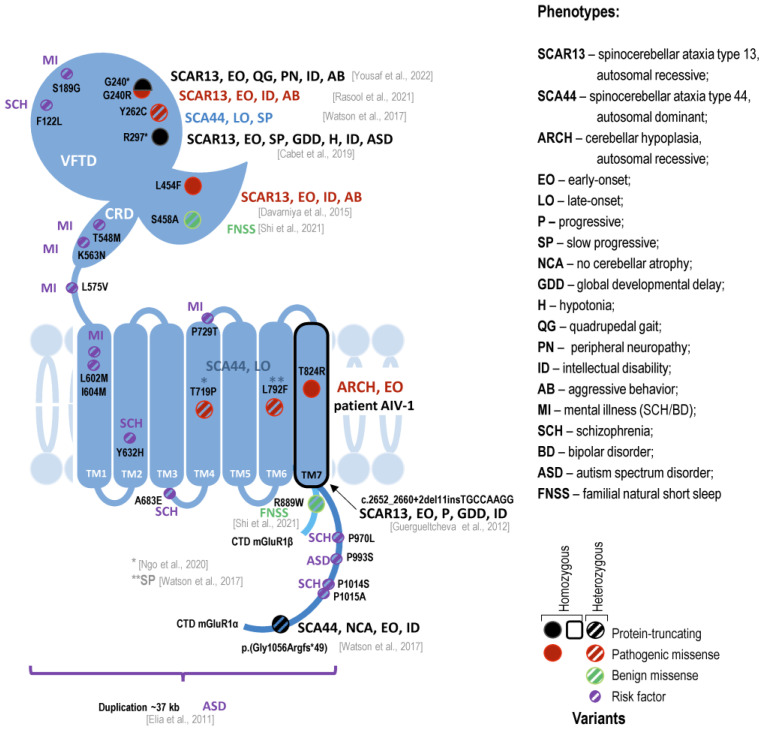
Schematic representation of mGluR1 and location of mutations associated with different phenotypes. mGluR1 is a dimeric receptor located on the postsynaptic membrane. The mGluR1 monomer consists of a large extracellular region, including the venus flytrap domain (VFTD), cysteine-rich domain (CRD), a region composed of 7 transmembrane domains (TM), and a C-terminal region (CDT) that differs in alpha (dark blue) and beta (light blue) isoforms [36]. The main phenotypes are indicated close to the mutations. SCAR13 represents the most severe form of the GRM1-associated diseases with development delay, speech and locomotion disturbance, coordination difficulties, tremors, oculomotor and pyramidal signs, different congenital anomalies, and intellectual impairment, often with aggressive behavior [32,40,41,42,43]. Heterozygous mutations can be the cause of SCA44 with the variable onset and disease severity [44,51,52]. Moreover, some mutations in the *GRM1* gene can affect sleep duration and efficiency [45]. Single nucleotide variations in *GRM1* gene can be a possible risk factors of schizophrenia, bipolar disorder, autism, and attention deficit hyperactivity disorder [46,47,48,49,50,53,54] Detailed information on clinical phenotypes is present in Table S1. Only references for a mutation linked with cerebellar pathology are indicated in Figure 3; references for single nucleotide variation risk factors are listed in Table S1.

Variants in the *GRM1* gene are extremely rare; to date, only a few pathogenic mutations leading to pathology of the cerebellum have been explored [23,24,37,38,39]. We analyzed all cases with defects in the *GRM1* gene reported to date and visualized all pathogenic genetic variants depending on the location of the protein domain (Figure 3). The clinical manifestation of the mutations varies from rare forms of movement disorder to different behavioral endophenotypes. Mutations in the *GRM1* gene result in both autosomal dominant spinocerebellar ataxia type 44 (SCA44; OMIM: 617691) and autosomal recessive spinocerebellar ataxia type 13 (SCAR13; OMIM: 614831). In the case of an autosomal recessive form, the course of the disease is severe, characterized by psychomotor developmental delay, intellectual disability, short stature, muscle hypotension, and severe impairment of motor functions throughout life; some patients are unable to move independently and speak [32,40,41,42,43]. A late-onset and mild disease progression have been described for the autosomal dominant form, although one patient had an early-onset with a more severe course and cognitive impairment [44]. The severity of the disease is likely related to the type of mutation in the gene. The mutations in the *GRM1* gene in two of the three published cases with autosomal recessive inheritance led to a disruption of the reading frame and truncation of the receptor [32,41]. Additionally, in a recent study, two rare polymorphic amino acid substitutions, one of which is located in the N-terminal extracellular and the other in the C-terminal intracellular domain of the isoform mGluR1β, were associated with a decrease in sleep duration and an increase in sleep efficiency [45]. In addition to these syndromes, there are also publications on the possible increased risk of schizophrenia, bipolar disorder, autism, and attention deficit hyperactivity disorder in the presence of a mutation in the *GRM1* gene [46,47,48,49,50]. Thus, the *GRM1* gene is an interesting example of the pleiotropic effects of mutations in one gene linked to both rare neurological and presumable neuropsychiatric disorders.

## 3. Materials and Methods

The study was approved by the Ethics Committee of the Research Center of Neurology with appropriate informed consent. Blood samples were collected from the patient, and DNA was extracted using a QIAamp DNA Blood Mini Kit (QIAGEN, Hilden, Germany). The exome library was prepared using a TruSeq Exome kit (Illumina, San Diego, CA, USA), and sequencing was performed using a NovaSeq 6000 System (Illumina, San Diego, CA, USA) by Genetico (Moscow, Russia). Bwa mem software was used to align raw reads to the human reference genome GRCh37/hg19 [55]. The mean depth of coverage of 71X was achieved. The variants were called using GATK tools [56,57,58]. Then, variants were annotated using VEP software [59]. Protein spatial structure was investigated using Phyre2 and AlfaFold [60,61]. The alignment of ortholog and paralog proteins was performed using the MEGA software and visualized using the GeneDoc software [61,62]. Validation was performed using Sanger sequencing on a 3730xl DNA Analyzer (Applied Biosystems, Foster City, CA, USA).

## 4. Conclusions

The new homozygous mutation is located in a highly conserved region in one of the transmembrane domains of the mGluR1 receptor and, in contrast to the previously described syndromes, has resulted in an autosomal recessive form of the congenital cerebellar ataxia, characterized by early-onset of the disease, delayed motor development, cerebellar hypoplasia, dysarthria, and ataxia with no obvious intellectual disability. In conclusion, the rare mutations in the *GRM1* gene contribute to a wide diversity of neurological phenotypes and may even be related to common neuropsychiatric illnesses.

## Data Availability

The data presented in this study are available on request from the corresponding author.

## Supplementary Materials

The following supporting information can be downloaded at: https://www.mdpi.com/article/10.3390/ijms24021551/s1.
