# Supplementary material for: Genetic Variant in GRM1 Underlies Congenital Cerebellar Ataxia with No Obvious Intellectual Disability
Source: Int J Mol Sci. 2023 Jan 12;24(2):1551. doi: 10.3390/ijms24021551 (PMC9865416; doi:10.3390/ijms24021551)
Supplement: Supplementary file 1 [file ijms-24-01551-s001.zip › Supplemental Figure S1.pdf]

Supplemental Figure

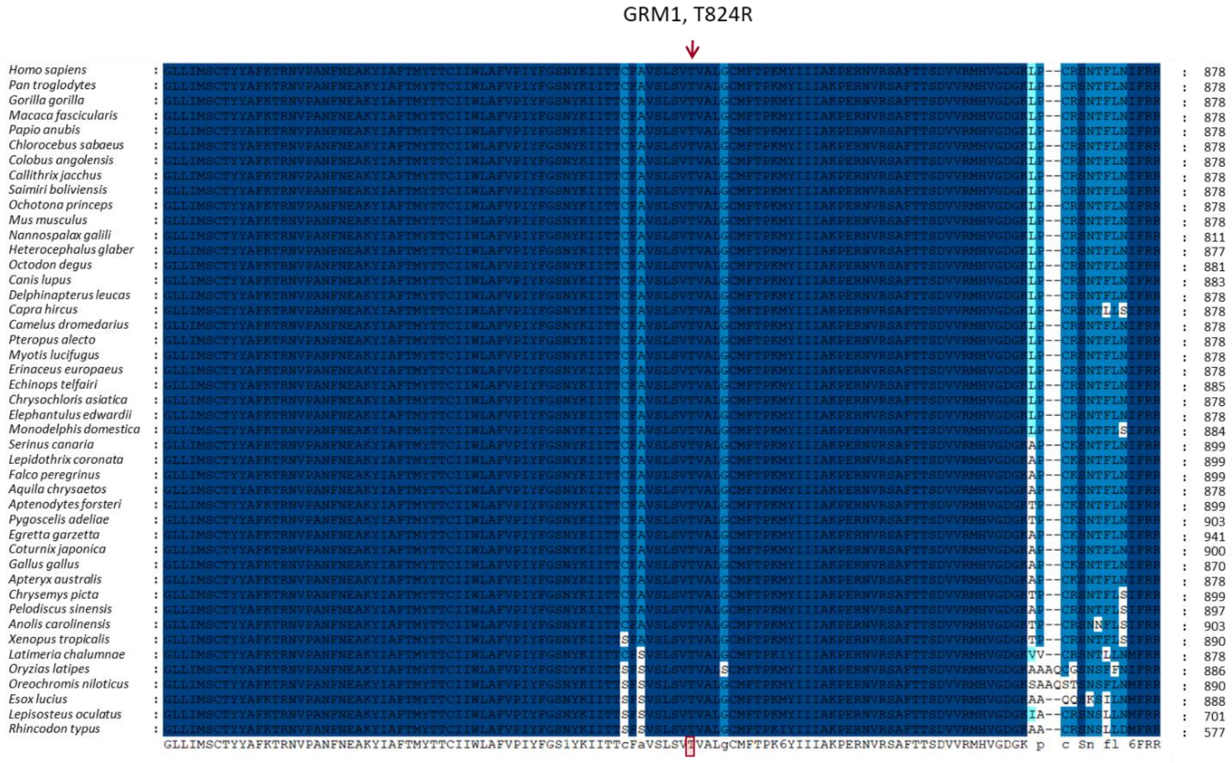

Figure S1. Extended evolutionary conservation analysis of mGluR1 regions harboring the amino acid substitution p.Thr824Arg across 45 species of vertebrates.
